# Supplementary material for: Digital immunoassay for biomarker concentration quantification using solid-state nanopores
Source: Nat Commun. 2021 Sep 9;12:5348. doi: 10.1038/s41467-021-25566-8 (PMC8429538; doi:10.1038/s41467-021-25566-8)
Supplement: Supplementary file 1 — Supplementary information. [file 41467_2021_25566_MOESM1_ESM.pdf]

Supplementary Information for

Digital Immunoassay for Biomarker Concentration Quantification using Solid-State Nanopores

*Liqun He[§], Daniel R. Tessier[§], Kyle Briggs, Matthaios Tsangaris, Martin Charron, Erin M. McConnell, Dmytro Lomovtsev, and Vincent Tabard-Cossa**

Department of Physics, University of Ottawa, Ottawa, Canada

§These authors contributed equally

*tcossa@uOttawa.ca

Table of Contents

Supplementary Notes 1. Design and Sequences for DNA Nanostructures 2
Supplementary Notes 2. DNA Nanostructure Assembly and Purification 4
Supplementary Notes 3. Dumbbell Event Detection 5
Supplementary Notes 4. Additional TSH Immunoassay Results 11
Supplementary Notes 5. Additional Nanopore Characterization of Nanostructures 18
Supplementary References.. 19

Supplementary Notes 1. Design and Sequences for DNA Nanostructures

Supplementary Table 1. Shooting Star and Dumbbell assembly oligo sequences

12-Arm relaxed core shooting stars probes (P-1 and P-2)			Bases (#)	GC Content (%)
**Star oligos**				
1	5'	AGGCACAGCTATAATAACGCAATCC/iSp18/TCTCCGGCCTCAAACTACTTTACCT	50	46
2	5'	AGGTAAAGTAGTTTGAGGCCGGAGA/iSp18/CCGAATGGAGTCTGTTCTCGACGCT	50	52
3+	5'	CAATTCTACTAATAGGAATTCGATTAGCGTCGAGAACAGACTCCATTCGG/iSp18/ACAAGTACGAA CCAACTTAGGACCT	75	43
4	5'	AGGTCCTAAGTTGGTTCGTACTTGT/iSp18/GGTCATCGTGGCGTACCATATACCT	50	48
5	5'	AGGTATATGGTACGCCACGATGACC/iSp18/TCTTCGATCTACCCGATAGGCTCCT	50	52
6	5'	AGGAGCCTATCGGGTAGATCGAAGA/iSp18/CGTACAGGTGTGACTTGAATTTGCT	50	48
7	5'	AGCAAATTCAAGTCACACCTGTACG/iSp18/AGTGTTAGAATACAACAAGCGACCT	50	42
8	5'	AGGTCGCTTGTTGTATTCTAACACT/iSp18/GCATCTCATACGACAGCATCCGACT	50	46
9	5'	AGTCGGATGCTGTCGTATGAGATGC/iSp18/TGAGCACGGAACTGTCAACCTTGCT	50	52
10	5'	AGCAAGGTTGACAGTTCCGTGCTCA/iSp18/CGTTCATTAAGATAAATCTGATCCT	50	42
11	5'	AGGATCAGATTTATCTTAATGAACG/iSp18/ACTATGACTGCTACATGCACTTCCT	50	38
12	5'	AGGAAGTGCATGTAGCAGTCATAGT/iSp18/GGATTGCGTTATTATAGCTGTGCCT	50	44
**Extension oligos common to P-1 and P-2**				
A	5'	AATCGAATTCCTATTAGTAGAATTGATGCCACCTCATCAGCTCGCGTCCG	50	46
B	5'	AACCTGTGTACGACTTAGATCTGTACGGACGCGAGCTGATGAGGTGGCAT	50	52
C	5'	TACAGATCTAAGTCGTACACAGGTTGCCTGAGTTGTGCGACGACGTCAGA	50	50
D	5'	CCGTAGCTGGACGTAGCGTGACCATTCTGACGTCGTCGCACAACTCAGGC	50	60
E	5'	ATGGTCACGCTACGTCCAGCTACGGGCAGAATTGGGAATCAACTGTTACA	50	50
**P-1 specific extension oligo**				
F	5'	GACGGTGTCTGGAAGTCTCACAGCATGTAACAGTTGATTCCCAATTCTGC	50	48
G	5'	TGCTGTGAGACTTCCAGACACCGTCCTTTAGTTGCATATAAGCTTCATGT	50	44
**P-2 specific extension oligo**				
F'	5'	CTACGTTCCTGGGTGACGAGCGTACTGTAACAGTTGATTCCCAATTCTGC	50	48
**Dumbbell Junction oligo**				
Junction	5'	/5PCBio/GTACGCTCGTCACCCAGGAACGTAGACATGAAGCTTATATGCAACTAAAG	50	46

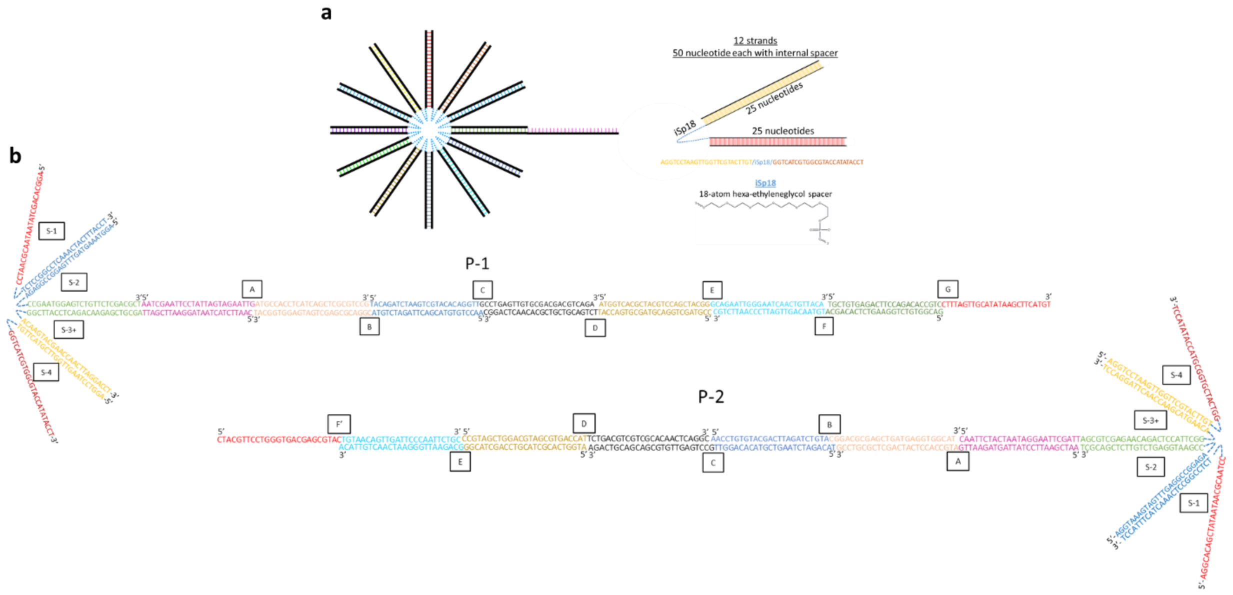

Supplementary Figure 1. Shooting star probes (P-1 and P-2) design. (a) Schematic representation of the star portion of the DNA probes. The star structure is composed of eleven 50 nucleotide oligos each containing a hexa-ethyleneglycol spacer (EG6) in the middle of the sequence after the first 25 nt. Oligo 3+ has an additional 25 nt ssDNA overhang (b) Schematic representation of the shooting star probes. The tail strands attach to the 25 nt ssDNA overhang present on strand (3+) upon which hybridize 7 staggering 50nt sequence staple strands (A-G) for the P-1 or 6 staggering 50nt sequence staple strands (A-F') for the P-2.

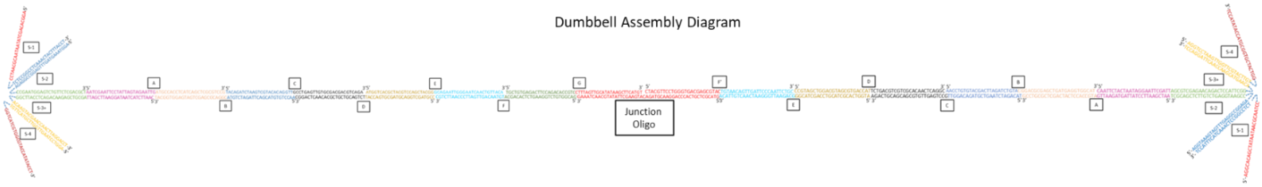

Supplementary Figure 2. Dumbbell assembly diagram. The shooting stars probes (P-1 and P-2) are joined together by a 50 nt junction oligo. Junction oligo halves are complementary to the respective 25 nt overhang present on the shooting star probe pair.

Supplementary Notes 2. DNA Nanostructure Assembly and Purification

A typical batch of shooting star assemblies can be visualized in Supplementary Figure 3. Initial shooting star assembly parameters were based on previous work from He *et al. 2019.*[1] Assembly of the shooting star probes were further optimized by varying the heat source (thermocycler vs heat block), incubation time, and oligo concentration. The optimized protocol is described in the Methods section of the main text. Supplementary Figure 3 lanes 4 and 5 show the crude assemblies of the P-1 and P-2 respectively. Due to the increase DNA nanostructure complexity, the top bright band corresponds to the fully formed shooting star probes. Even though it is the dominant band, it only makes up 20-30% of the total population, as measured through gel electrophoresis band density estimates. This leaves a significant portion of the nanostructures as misassembled products that have the potential to cause false positives and false negatives during nanopore sensing. Because of this, all nanostructures employed for nanopore experiments are purified by gel extraction (described in the main text Methods section), which increases the fraction of correct shooting star assemblies to 80-90%. Pure products can be visualized in Supplementary Figure 3 lanes 2 and 3. Agarose gel extraction yielded better recovery after purification, however, the gel introduced further which caused pore clogging.

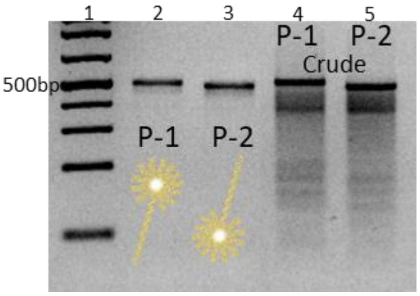

Supplementary Figure 3. Agarose gel (2%) of shooting star probes P-1 and P-2 assemblies and purification. Lane 1: GeneRuler 1kb Plus DNA Ladder (ThermoFisher Scientific, SM1331), from bottom 75, 200, 300, 400, 500, 700, 1000, 1500, 2000, 3000, 4000, 5000, 7000, 10000, and 20000 bp, lane 2: gel extracted P-1, lane 3: gel extracted P-2, lane 4: crude P-1 and lane 5: crude P-2.

Supplementary Notes 3. Dumbbell Event Detection

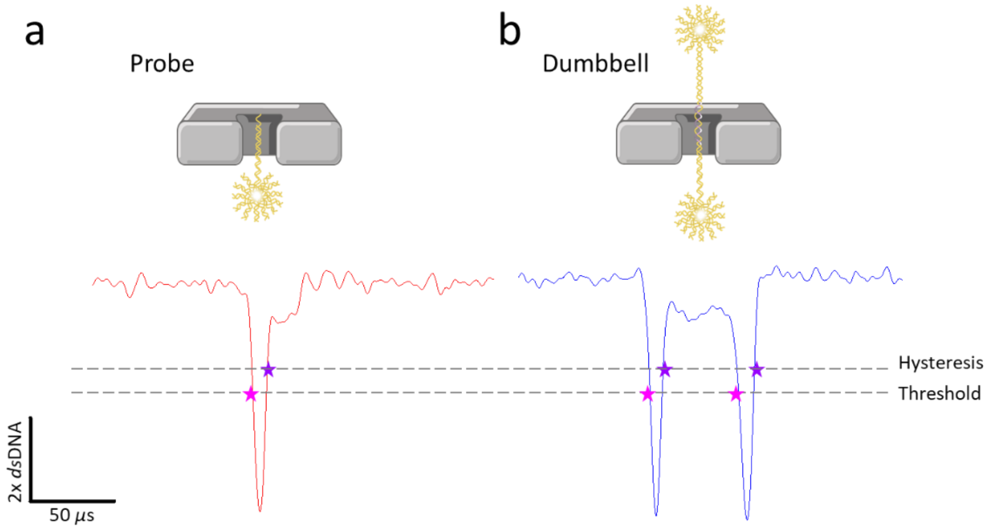

Supplementary Figure 4. Dumbbell detection using threshold crossing scheme. (a) A shooting star probe event registers 2 intra-crossings. (b) A dumbbell event registers 4 intra-crossings. The threshold is set to 2.5x dsDNA unless otherwise specified. To avoid triggering on noise, the threshold that indicates the return to baseline is closer to the baseline than the threshold that indicates the start of the deep part of the event (hysteresis).

Translocation events in the current recordings are analyzed using a custom version of the CUSUM+ algorithm[2,3], and a simple threshold-crossing algorithm is used to label events as being due to dumbbell or shooting star probe translocation. Data analysis is performed with a digital low-pass Bessel filtered at 200 kHz. A shooting star probe event contains two threshold crossings and a dumbbell event contains four threshold crossings. The threshold to indicate the start of a deeper sub event is set to 2.5x the average linear dsDNA blockage for that pore, normalized to the local baseline current, while the threshold to indicate the end of that sub event is set to 6x baseline standard deviations.

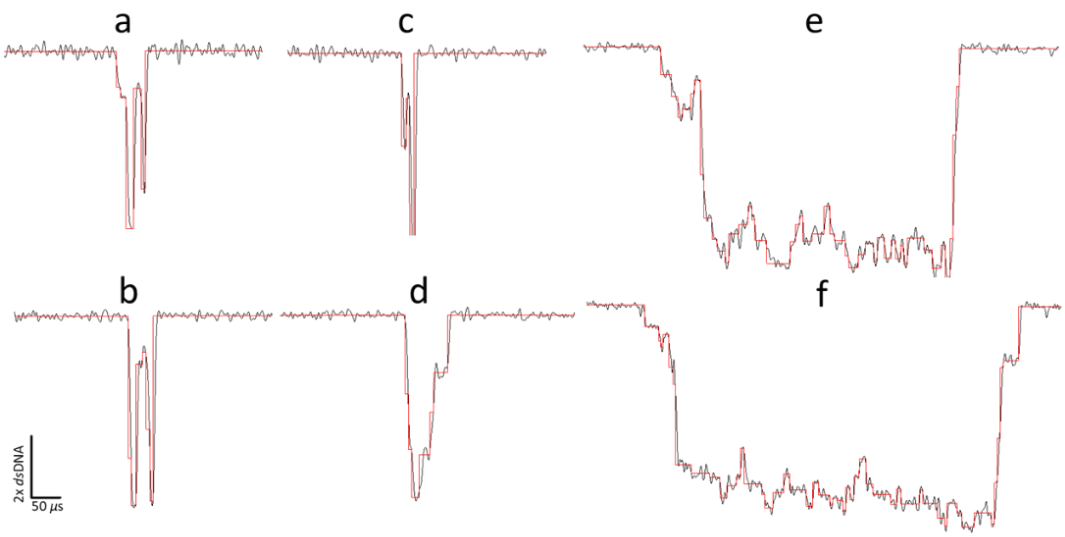

Supplementary Figure 5. Example current traces of false positive events. (a-b) Current traces showing two distinct peaks, representing shooting star probe pairs connected by a dsDNA, indicating formation of DB due to non-specific binding. (c) Example current trace of an event with one peak significantly shallower than the other, representing a shooting star event with a folded extension tail, resulting in false positive. (d-f) Current traces of shooting stars performed using an 11 nm pore, with an applied bias of 100 mV.

The progression of the dumbbell assembly (10 nM junction ssDNA to 20 nM shooting star probes occurs quickly from 30 minutes to 4 hours, at which point it starts to level off, reaching a plateau at the 2-day mark and remaining at the plateau until beyond the 10-day mark. Interestingly, we do not observe any increase in dumbbell fraction during the nanopore sensing experiments even at short incubation times. Evidently, the dumbbell formation does not take place at an appreciable rate in the nanopore running buffer of 3.2 M LiCl, for the “0.5-hr” experiment in particular in Supplementary Figure 6c (red). The dumbbell fraction stabilizes to 17 % during the nanopore sensing time of 20 minutes (total of 50 minutes since the start of incubation) rather than increasing towards the 25 % observed in to the “1-hr” experiment. Nanopore sensing was not performed on samples incubated longer than 10 days. Reductions in the required incubation time can be achieved by ensuring an excess of shooting stars over junctions.

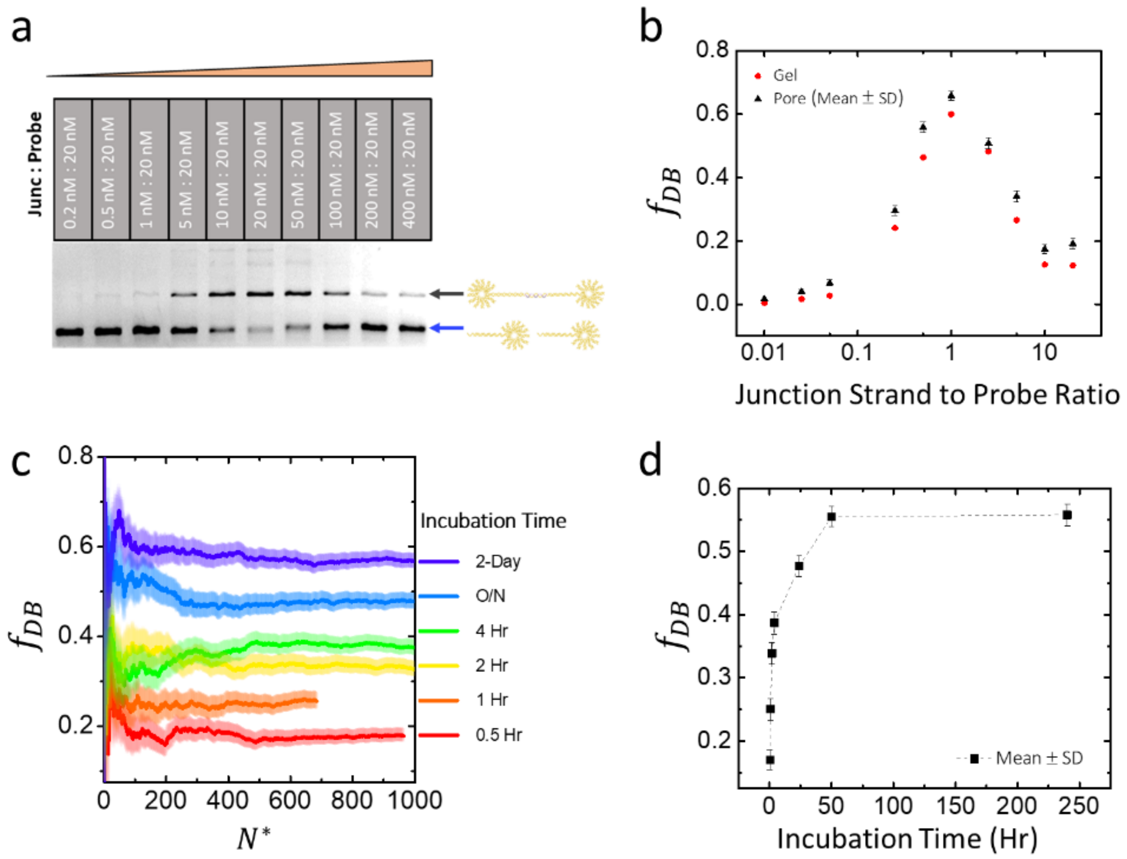

Supplementary Figure 6. Dose response and time dependence of dumbbell assembly. (a) Gel electrophoresis (2% agarose) of dumbbells formation and remaining shooting star probes as junction concentration increases. (b) Comparison of dose response with junction concentration ranging from 0.2 nM to 400 nM (junction to shooting star probes ratio of 0.01:1 to 20:1) between nanopore sensing with $N = \sim 1100$ single-molecule events at each concentration and gel band densitometry. (c) The time response of dumbbell events fraction f_{DB} for incubation time of 30 minutes to 2 days. (d) Dumbbell events fraction f_{DB} as a function of incubation time. The sample (10 nM junction ssDNA and 20 nM shooting star probes) is prepared in 1xTAEMg with a total volume of 35 μL, and 5 μL is sequentially taken from the sample and subjected to nanopore sensing after incubations of 0.5, 1, 2, 4, 24 (O/N), and 48 hours. The error bars represent one standard deviation following Equation 3 of the main text. All experiments are performed using an 11.3 nm pore, with an applied bias of 100 mV and the intra-crossings threshold set to 2.5x dsDNA.

Even when gel extracted purified dumbbell, 100% positive events is not attainable. Supplementary Figure 7a shows a scatter plot of the purified dumbbell in which a small fraction of the shooting star or non-dumbbell events is still present. The threshold is set to multiples of the standard deviation of the local segment of the current baseline, making it a dynamic value which depends on the baseline value and noise. Supplementary Figure 7b shows that the standard deviation of the

baseline is indeed fluctuating. Supplementary Figure 7c shows the gel electrophoresis results obtained using purified dumbbells, confirming the existence of impurities.

Two different batches of dumbbells are gel extracted and sensed on two different pores, as shown in Supplementary Figure 7d-e, registering 81 ± 2 % and 84 ± 3 % dumbbells (instead the expected 100 %) using 13.6 and 12.7 nm pores. Potential contributing factors specific to the nanopore, include changes in baseline current, over-/under-crossing of the threshold and potential mechanical damage during translocation. In the case of over-crossing, the "dip" is higher than the set threshold (2.5x dsDNA), as shown in Supplementary Figure 7g, resulting in the false negative event, while in the case of under-crossing, one of the stars in a dumbbell molecule translocates too fast and is not fully resolved by the recording device, resulting in one of the peaks having a blockage lower than the set threshold (2.5x dsDNA), as shown in Supplementary Figure 7h (false negative). Another potential source of error would be partial degradation of the fully formed dumbbell nanostructure post purification. Evidence of this can be seen on the agarose gel (Supplementary Figure 7c) as multiple faint bands below the purified dumbbell bright band.

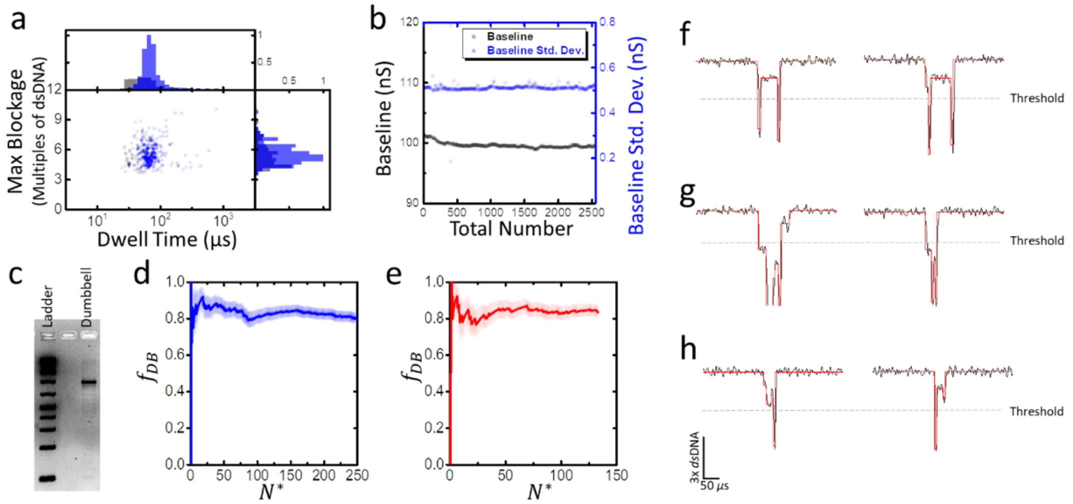

Supplementary Figure 7. Purified dumbbell translocation characteristics. (a) Scatter plot and projected histograms of dwell time distribution and maximum blockage distribution of purified dumbbell (DB) using a 13.6 nm pore. (b) Baseline (black, left axis) and the standard deviation of the baseline (blue, right axis) *versus* total number of events of the purified DB profile shown in (a). (c) Gel electrophoresis of purified DB batch 1 using 2 % Agarose gel in 0.5x TBE with an applied voltage of 100 V. Lane 1: GeneRuler 1kb Plus DNA Ladder (ThermoFisher Scientific, SM1331), from bottom 75, 200, 300, 400, 500, 700, 1000, 1500, 2000, 3000, 4000, 5000, 7000, 10000, and 20000 bp. (d-e) Time response of purified DB batch 1 and batch 2 using a 13.6 nm

pore (blue) and a 12.7 nm pore (red) respectively. (f-h) Example current traces (black) and their fits (red) representing good crossing, over crossing, and under crossing. All experiments are performed in 3.2 M LiCl with an applied bias of 100 mV.

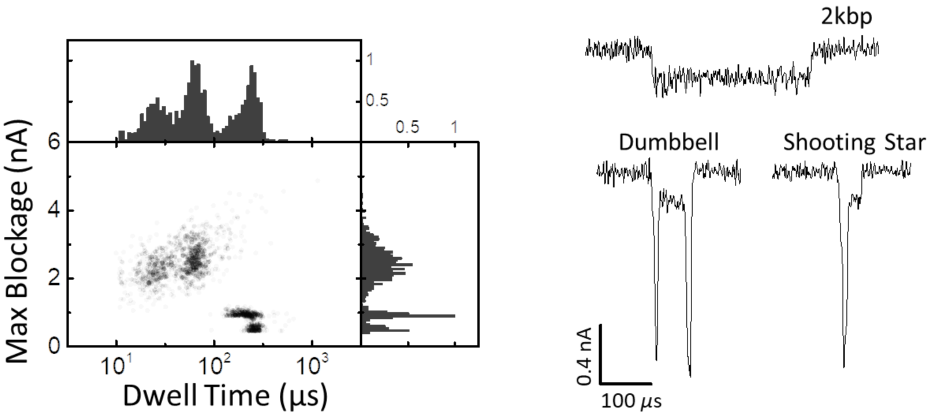

Supplementary Figure 8. Scatter plot and projected histograms of dwell time distribution and maximum blockage distribution of a mixture of nanostructures (shooting star, dumbbell, and 2kbp dsDNA) and their representative current traces. The nanopore experiment is performed using an 12 nm pore, in 3.2 M LiCl, with an applied bias of 100 mV and the intra-crossings threshold set to 2.5x dsDNA.

Supplementary Figure 8 shows a scatter plot of maximum blockage (in nA) *versus* dwell time of shooting star, dumbbell, and 2kbp dsDNA. In the main article, we use the unit of dsDNA which refers to the current blockage level of 2kbp dsDNA, all experiments were done with linear 2kbp DNA in the mixture, they were added as a molecular ruler to help reduce pore-to-pore and experiment-to-experiment variance in current blockage level, to ensure consistency in the threshold-crossing algorithm.

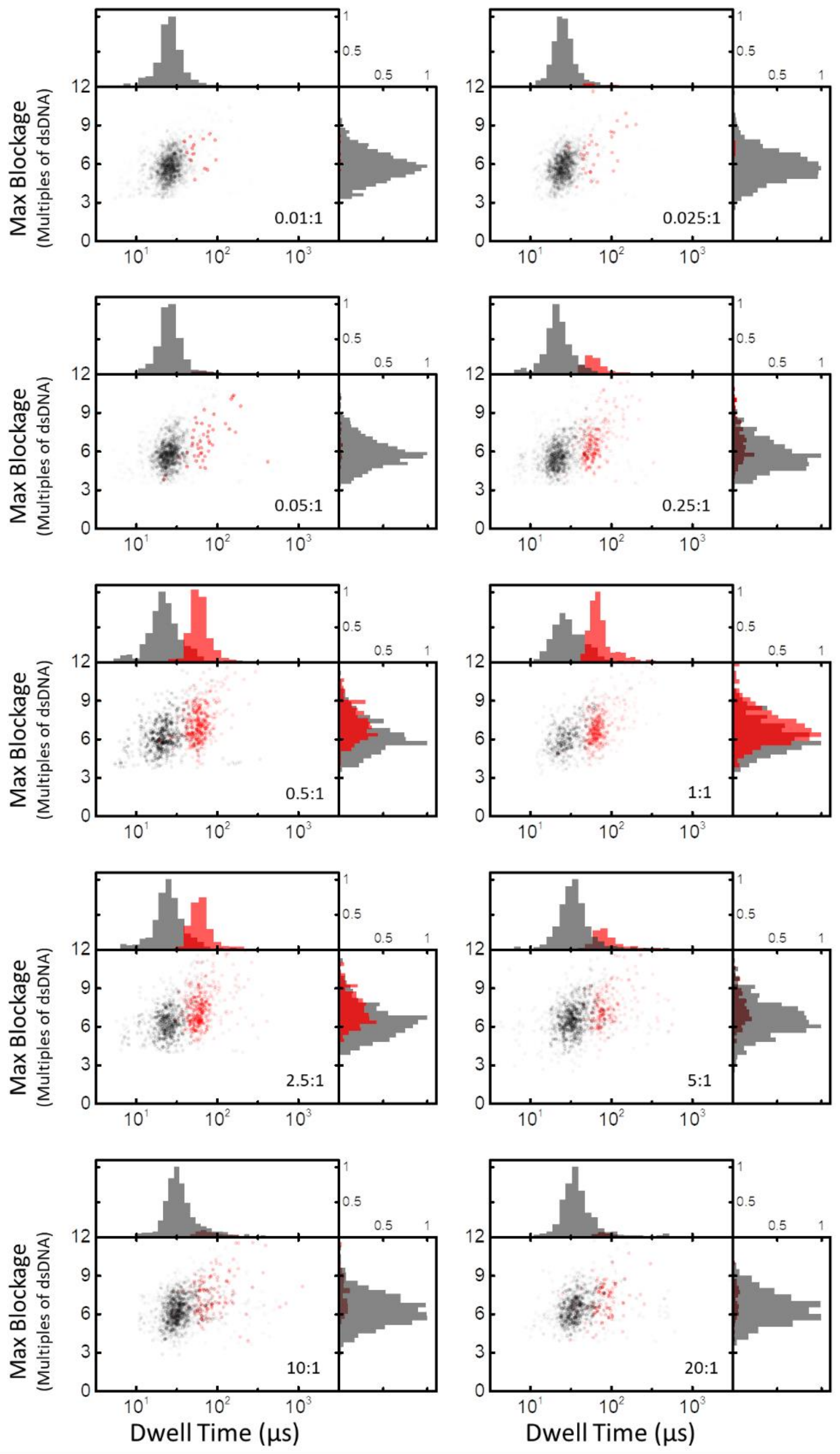

Supplementary Figure 9. Scatter plots and histograms of maximum blockage *versus* dwell time (log scale) of dose response in Figure 3, of junction strand to shooting star ratio from 0.01:1 to 20:1, showing 1639, 1524, 1268, 1337, 917, 882, 964, 1019, 1270, and 853 single-molecule events, respectively. Dumbbell events (red) are separated from shooting star events (dark grey) using the threshold-crossing algorithm.

Supplementary Notes 4. Additional TSH Immunoassay Results

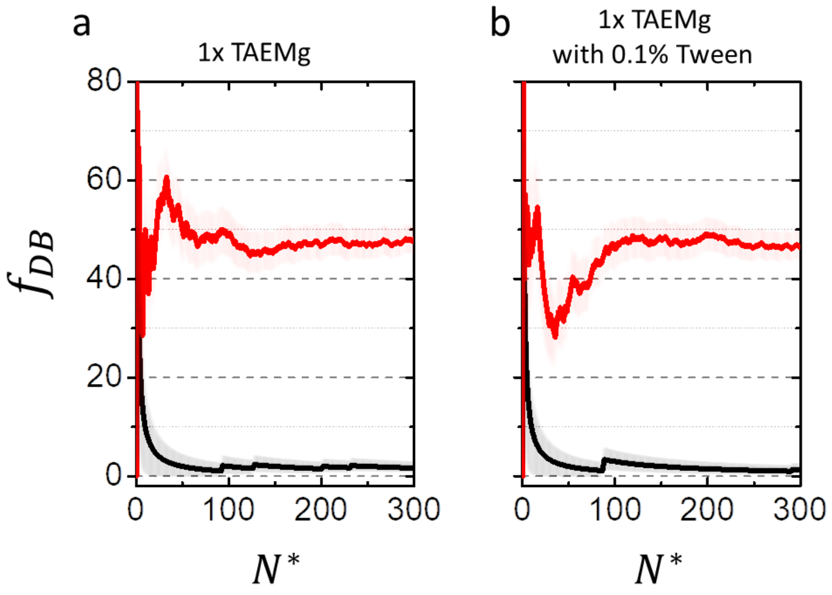

Supplementary Figure 10. Assay buffer test and the effect on dumbbell assembly and nanopore detection. Time response of dumbbell formation with (red) and without (black) the junction ssDNA in (a) 1x TAEMg (b) 1x TAEMg with 0.1% tween 20. All four experiments are performed in 3.2 M LiCl with applied bias of 100 mV using an 11 nm pore, a 20 nM to 20 nM junction to shooting star ratio was used, with the threshold set to 2.5x dsDNA.

The use of tween-20 in the final steps of the assay during release of the junction strand significantly reduces assay components sticking to the tube walls. To ensure that the surfactant used during the assay steps does not affect nanopore performance, a set of comparison experiments are carried out. The samples are: 1) Mixture of equimolar P-1 and P-2 at 20 nM in 1xTAEMg. 2) Mixture of equimolar P-1 and P-2 at 20 nM with 20 nM junction ssDNA. 3) Mixture of equimolar P-1 and P-2 at 20 nM in 1xTAEMg plus 0.1 % tween-20. 4) Mixture of equimolar P-1 and P-2 at 20 nM with 20 nM junction ssDNA plus 0.1 % tween. All four samples are sensed on an 11 nm pore under the same experimental conditions. A background of 1.7% and 1.4% dumbbell events are detected for samples 1 and 3, while 47.1% and 46.7% are detected for samples 2 and 4, as shown in Supplementary Figure 10. We observe that 0.1% tween has no effect the assembly of dumbbells, and it does not induce additional background, as identical results are reported in Supplementary

Figure 10 with and without 0.1% tween-20. Furthermore, 0.1% tween has no measurable effect on nanopore sensing performance.

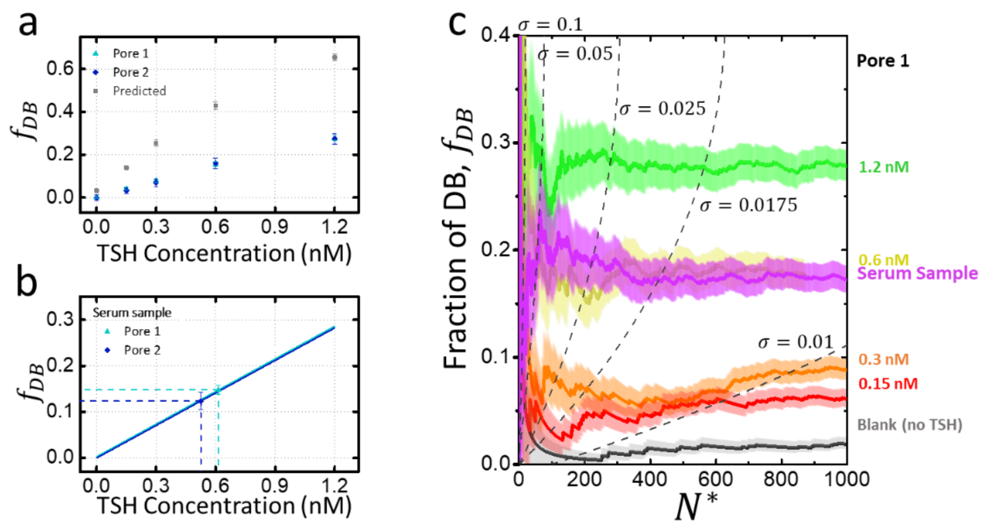

Supplementary Figure 11. TSH assay calibration curve and TSH serum sample quantification using solid-state nanopores with a different batch of reagents. (a) TSH calibration curve concentration, blank 0, 0.15 nM, 0.30 nM, 0.60 nM, and 1.2 nM, repeated using pore 1 (12.5 nm) and pore 2 (14 nm). (b) 0.48 nM TSH spiked serum on the same pores. (c) The time response of the assay calibration points, fraction of dumbbells (DB) events as a function of effective event count detected using a 10 nm pore. The colored bands represent one standard deviation. The end points of each calibration point with two standard deviations are projected on the right. The time response of serum sample spiked with 0.48 nM TSH, repeated using pore 1 (12.5 nm). The probes P-1 and P-2 are fixed at 20 nM. All experiments are performed in 3.2 M LiCl in a mixture with 2kbp dsDNA standard with an applied bias of 100 mV and the intra-crossings threshold set to 2.5x dsDNA.

The TSH assay repeats are conducted using a different batch of reagents on pore 1 (12.5 nm) and pore 2 (14 nm) as shown in Supplementary Figure 11. Following the assay scheme, the ssDNA junctions released from the sandwich assay corresponding to 0, 0.15, 0.3, 0.6 and 1.2 nM TSH concentrations are collected and mixed with shooting star pairs at a fixed concentration of $c_1 = c_2 = 20$ nM. A 5-point calibration curve is then constructed, as shown in Supplementary Figure 9. Backgrounds of 2% and 5% positive events are detected in the blank sample (subtracted from calibration points) using the 12.5 nm and 14 nm pore, respectively. A serum sample spiked with 0.48 nM TSH was then sensed on the 2 pores, measuring 17 $\pm$1% and 17 $\pm$2% positive events

respectively. Since the calibration curve shows a linear dose response, we interpolate a target to probe ratio of 0.51 and 0.44, corresponding to 10.2 nM and 8.7 nM junctions in the mixture with shooting star probes and predicting the initial target TSH concentration in the assay to be 0.61 ±0.03 and 0.53 nM ±0.08, respectively. Supplementary Figure 11c shows that at least 67, 113, 196 and 279 effective event counts are required to obtain an absolute uncertainty of σ_f = 0.025 (2.5%), for TSH concentrations of 0.15 nM, 0.3 nM, 0.6 nM and 1.2 nM, respectively.

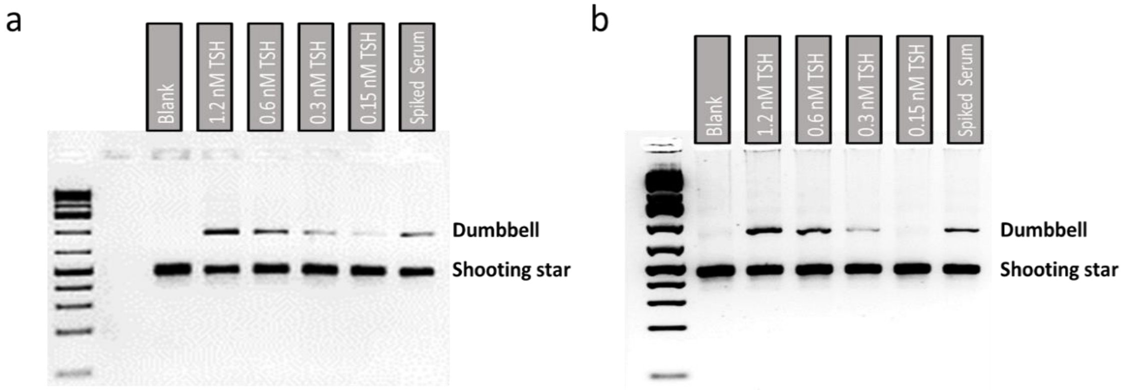

Supplementary Figure 12. Dumbbell formation as a function of target protein concentration using 2 % Agarose gel. (a-b) TSH assay samples from left to right: blank (no TSH), 1.2, 0.6, 0.3, 0.15 nM TSH, and 0.48 nM TSH spiked serum sample. (a) Same samples as the nanopore TSH assay shown in Figure 4 of the main article. Lane 1: GeneRuler 1kb Plus DNA Ladder (ThermoFisher Scientific, SM1331), from bottom 75, 200, 300, 400, 500, 700, 1000, 1500, 2000, 3000, 4000, 5000, 7000, 10000, and 20000 bp.

Supplementary Figure 12 shows gel electrophoresis of the both TSH assay demonstrated in main text Figure 4, and in Supplementary Figure 11, confirming our observations from the nanopore sensing in which we can observe 1) the blank, no visible dumbbell band (nanopore false positive, signal to noise of dumbbell like DNA nanostructures), 2) 1.2 nM to 0.15 nM TSH calibrators, a decreasing amount of dumbbell products and 3) the 0.48 nM TSH spiked serum, the dumbbell band intensity appears as it should between the 0.6 nM and 0.3 nM calibrators bands intensities.

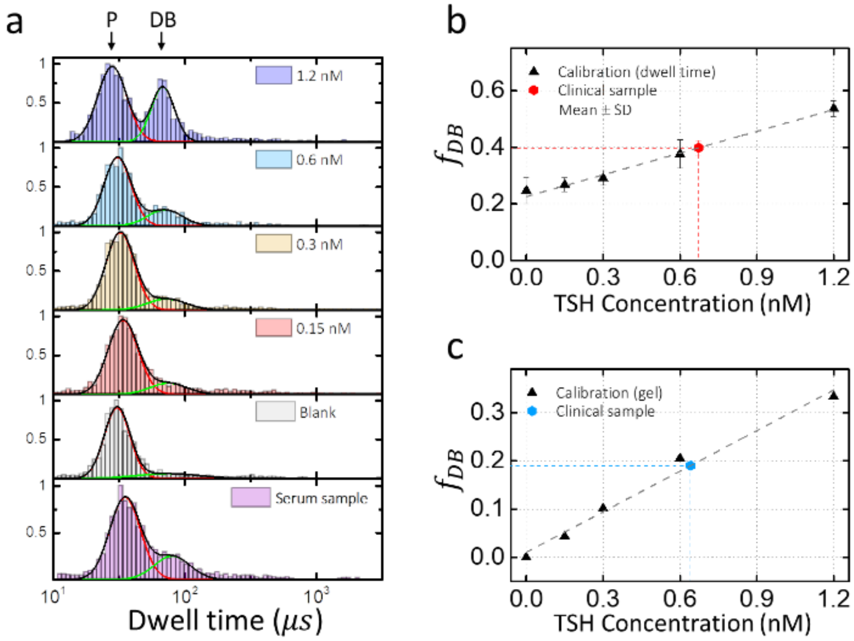

Supplementary Figure 13. Additional analysis of TSH assay. (a) Histograms of nanopore dwell time distribution. Calibration curves of TSH concentration of blank, 0.15, 0.3, 0.6, and 1.2 nM, are constructed using (b) Gaussian fits of dwell time distribution, with $N = 1251$, 1247, 1750, 1301, 1239, and 1522 single-molecule events, error bars represent one standard deviation from the Gaussian fits, and (c) Relative gel electrophoresis band intensity. Serum sample spiked with 0.48 nM TSH are measured and plotted using both methods. All nanopore experiments are performed using a 10 nm pore in 3.2 M LiCl with an applied bias of 100 mV. Gel electrophoresis is performed using 2% Agarose in 0.5x TBE buffer at 100 V.

Although the use of threshold crossings using nanopores has shown great consistency and reproducibility, we also compare to a few other methods of quantification. Here, besides using the threshold technique as presented in main text Figure 4, we extracted DB fraction by Gaussian fitting the dwell time distribution and relative gel electrophoresis band intensity for the same data set. Supplementary Figure 13a shows the histogram of dwell time distributions of TSH concentration of blank, 0.15, 0.3, 0.6, and 1.2 nM, and serum sample spiked with 0.48 nM TSH. A calibration curve of dumbbell formation *versus* TSH concentration is constructed by comparing dwell time distribution, as shown in Supplementary Figure 13b, the serum sample spiked with 0.48 nM TSH is then plotted and extrapolated from the fit, measuring 0.67 nM. Similarly, by comparing gel electrophoresis intensities of bands from Supplementary Figure 12 for different TSH concentrations, the same 5-point calibration curve is constructed as shown in Supplementary

Figure 13c. The serum sample spiked with 0.48 nM TSH is performed on the same gel, extrapolating the DB fraction obtained from gel electrophoresis in Supplementary Figure 13c, it measures 0.64 nM. The alternative methods of dwell time distribution analysis and gel band intensity analysis predict TSH initial concentrations of 0.67 and 0.64 nM, comparable to the nanopore results obtained using threshold crossing (0.54, 0.61, and 0.59 nM using 10, 11.5, and 12 nm pores, respectively, as shown in Figure 4).

Although the dwell time analysis yielded similar results and gel band analysis yield similar results, the two alternative methods have their limitations. The nanopore-based dwell time distribution analysis relies on the correct fitting of the histograms, and this can be particularly troublesome for lower concentrations. The calibration curve in Supplementary Figure 13b levels off at low concentrations and the dose response appears to be no longer in a linear regime, making the dwell time analysis method suffer from reductions of the dynamic range, limit of detection, and sensitivity compared to the threshold crossing method (Figure 4). Gel band intensity analysis provides accurate and consistent results, but requires a relatively large amount of sample for lower concentration limits compared to threshold crossing. Meanwhile, nanopore-based threshold crossing method can reliably detect and distinguish dumbbell from shooting star on a single-molecule basis at low concentrations, which practically allows for real time analysis as it only requires a few hundred single molecule events (5 minutes of nanopore sensing) to generate statistically meaningful results.

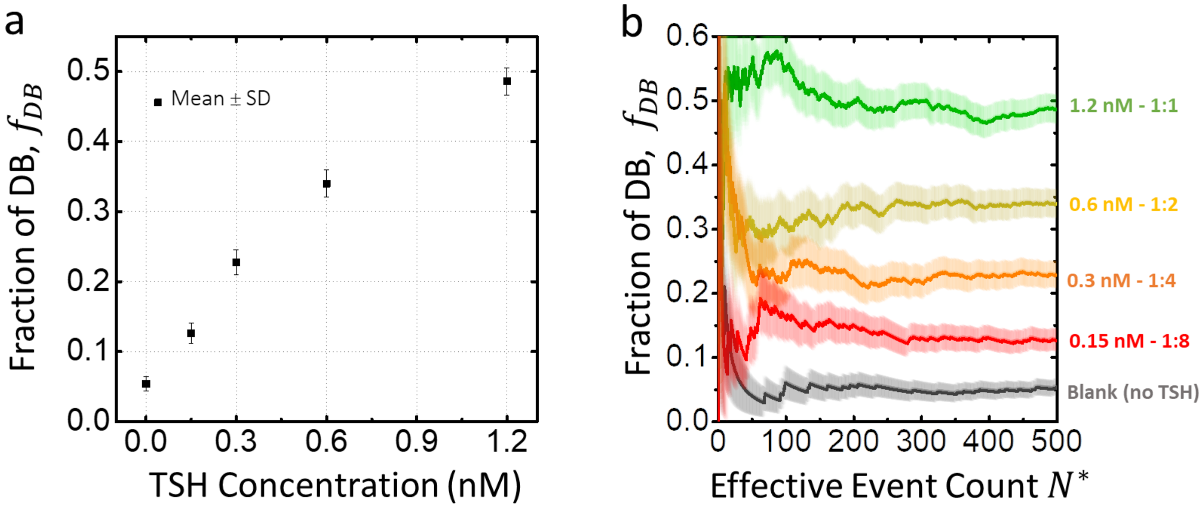

Supplementary Figure 14. Additional TSH measurements in human serum sample. a) TSH calibration curve concentration, 0 (blank), 0.15 nM, 0.3 nM, 0.6 nM, and 1.2 nM, with $N = 973$, 936, 887, 821, and 1337 single-molecule events. The error bars represent one standard deviation following Equation 3 of the main text. b) Fraction of dumbbell events as a function of the effective event count $N^* = N_{DB} + N_P/2$ detected for dumbbells and probes. Experiments are performed in 4x diluted human serum and sensed using a 12 nm pore in 3.2 M LiCl at 100 mV. Intra-crossings threshold is set 2.5x dsDNA, all experiments are low-pass Bessel filtered at 200 kHz for analysis.

Additionally, we repeated our nanopore assay and performed five additional measurements of rTSH in human serum sample from 0.15 nM to 1.2 nM using a 12 nm pore, as shown in Supplementary Figure 14. We performed a homebrew ELISA assay employing the same workflow and reagents as our nanopore assay to validate our method. To do so, we replaced the biotinylated junction strand with a commercial biotinylated-HRP. Compared to the detection range (~0.1 – 70 pM) of the commercially available ELISA for TSH (ThermoFisher Scientific), our homebrew ELISA preformed as expected, allowing us to detect low (0.8 pM), medium (3.2 pM) and high (12.7 pM) concentrations that corresponded with our nanopore assay as shown in Supplementary Figure 15. Note that the homebrew ELISA was carried out using the same nanopore assay workflow, which was not optimized for optical readouts.

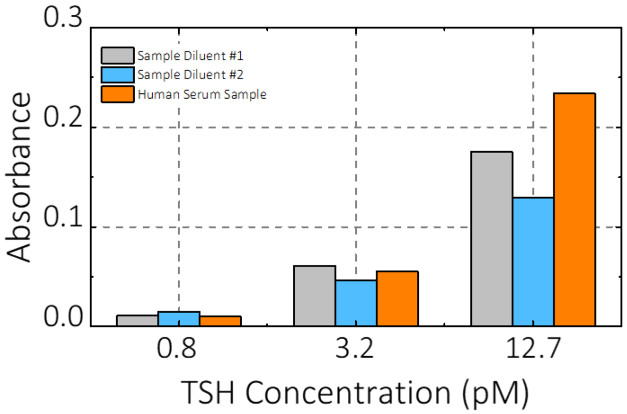

Supplementary Figure 15. Validation of assay components by comparison of signal in sample diluent and low-mid-high values in human serum at nanopore assay concentrations. Commercial biotinylated HRP was used to produce an optical readout, with same assay components and steps scaled down to volumes compatible with a 96 well plate and a 24 magnet base. The values presented have been corrected by subtracting the absorbance of the blank. Briefly, primary antibody coated magnetic beads were washed with sample diluent then combined with TSH in either sample diluent (SD) or 1 in 4 diluted human serum sample, and allowed to incubate for 1.5 h at room temperature (RT) with gentle agitation. The beads were washed three times for 5, 10, then 15 minutes, then were incubated with the secondary antibody in sample diluent for 1 h at RT. Following 3 quick washes, the beads were incubated with biotinylated HRP (ThermoFisher Scientific, 432040) for 15 min at RT. Following a final 3 quick washes the presence of TSH was indicated by incubation with TMB ELISA Substrate (fast kinetics ab171524) for 17.5 min then stopped using 450 nm Stop Solution for TMB Substrate (Abcam,ab171529). The absorbance at 450 nm was read using a BioTek EPOC 2 spectrophotometer (Biotek, BTEPOCH2).

Supplementary Notes 5. Additional Nanopore Characterization of Nanostructures

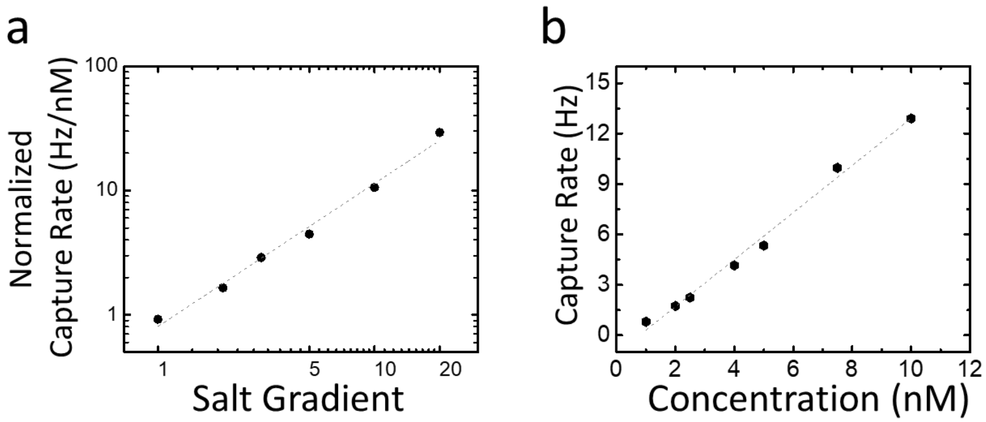

Supplementary Figure 16. Nanopore capture rate analysis of shooting star probes. (a) Normalized capture rate of shooting stars as a function of salt gradient, with C_{cis} = 0.18, 0.36, 0.72, 1.2, 1.8, and 3.6 M LiCl and C_{trans} fixed at 3.6 M LiCl. (b) The capture rate of shooting star as a function of shooting star concentration. All experiments are performed using an 8.5 nm pore with an applied bias of 200 mV.

Following the previous work in salt asymmetry,[1,4] the capture rate of the shooting star can be improved while increasing the translocation time and maintaining a high SNR. The normalized capture rate is increased from 0.9 Hz/nM in symmetric salt (C_{cis} = C_{trans} = 3.6 M LiCl) to 29 Hz/nM with the use of a 20x salt gradient (C_{cis} = 0.18 M LiCl and C_{trans} = 3.6 M LiCl) using an 8.5 nm pore as shown in Supplementary Figure 16a. Moreover, Supplementary Figure 16b shows the capture rate of shooting star as a function of its concentration, the capture rate of shooting star exhibits a linear response to the concentration of the molecule.

Supplementary References

1. He, L., Karau, P. & Tabard-Cossa, V. Fast capture and multiplexed detection of short multi-arm DNA stars in solid-state nanopores. *Nanoscale* **11**, 16342–16350 (2019).

2. Briggs, K. Solid-State Nanopores: Fabrication, Application, and Analysis. PhD Thesis, University of Ottawa (2018).

3. Forstater, J. H. *et al.* MOSAIC: A modular single-molecule analysis interface for decoding multistate nanopore data. *Anal. Chem.* **88**, (2016).

4. Wanunu, M., Morrison, W., Rabin, Y., Grosberg, A. Y. & Meller, A. Electrostatic focusing of unlabelled DNA into nanoscale pores using a salt gradient. *Nat. Nanotechnol.* **5**, 160–5 (2010).
