# Supplementary material for: Digital immunoassay for biomarker concentration quantification using solid-state nanopores
Source: Nat Commun. 2021 Sep 9;12:5348. doi: 10.1038/s41467-021-25566-8 (PMC8429538; doi:10.1038/s41467-021-25566-8)
Supplement: Supplementary file 2 — Reporting summary. [file 41467_2021_25566_MOESM2_ESM.pdf]

nature portfolio

Corresponding author(s): Vincent Tabard-Cossa

Last updated by author(s): Aug 9, 2021

Reporting Summary

Nature Portfolio wishes to improve the reproducibility of the work that we publish. This form provides structure for consistency and transparency in reporting. For further information on Nature Portfolio policies, see our Editorial Policies and the Editorial Policy Checklist.

Statistics

For all statistical analyses, confirm that the following items are present in the figure legend, table legend, main text, or Methods section.

n/a	Confirmed	
☐	☒	The exact sample size (n) for each experimental group/condition, given as a discrete number and unit of measurement
☐	☒	A statement on whether measurements were taken from distinct samples or whether the same sample was measured repeatedly
☒	☐	The statistical test(s) used AND whether they are one- or two-sided<br>*Only common tests should be described solely by name; describe more complex techniques in the Methods section.*
☐	☒	A description of all covariates tested
☐	☒	A description of any assumptions or corrections, such as tests of normality and adjustment for multiple comparisons
☐	☒	A full description of the statistical parameters including central tendency (e.g. means) or other basic estimates (e.g. regression coefficient) AND variation (e.g. standard deviation) or associated estimates of uncertainty (e.g. confidence intervals)
☒	☐	For null hypothesis testing, the test statistic (e.g. F, t, r) with confidence intervals, effect sizes, degrees of freedom and P value noted<br>*Give P values as exact values whenever suitable.*
☒	☐	For Bayesian analysis, information on the choice of priors and Markov chain Monte Carlo settings
☒	☐	For hierarchical and complex designs, identification of the appropriate level for tests and full reporting of outcomes
☒	☐	Estimates of effect sizes (e.g. Cohen's d, Pearson's r), indicating how they were calculated

Our web collection on statistics for biologists contains articles on many of the points above.

Software and code

Policy information about availability of computer code

Data collection	LabView for nanopore fabrication, Chimera Instruments MatLab program for nanopore recording, and Azure Biosystems gel imaging software.
Data analysis	ImageJ was used for band intensity in gel images, OriginLab for plotting data, and all other data analysis was done using in-house program available at https://www.github.com/shadowk29/CUSUM

For manuscripts utilizing custom algorithms or software that are central to the research but not yet described in published literature, software must be made available to editors and reviewers. We strongly encourage code deposition in a community repository (e.g. GitHub). See the Nature Portfolio guidelines for submitting code & software for further information.

Data

Policy information about availability of data

All manuscripts must include a data availability statement. This statement should provide the following information, where applicable:

- Accession codes, unique identifiers, or web links for publicly available datasets
- A description of any restrictions on data availability
- For clinical datasets or third party data, please ensure that the statement adheres to our policy

Raw data supporting the immunoassay results within this paper are accessible via Federated Research Data Repository: https://doi.org/10.20383/102.0483. Any other data including validation and repeated experiments are available from the corresponding author upon reasonable request due to limited storage available. All processed data are provided in the Source Data file.

Field-specific reporting

Please select the one below that is the best fit for your research. If you are not sure, read the appropriate sections before making your selection.

- [x] Life sciences
- [ ] Behavioural & social sciences
- [ ] Ecological, evolutionary & environmental sciences

For a reference copy of the document with all sections, see nature.com/documents/nr-reporting-summary-flat.pdf

Life sciences study design

All studies must disclose on these points even when the disclosure is negative.

Sample size	No formal methods were used, but data points for the dumbbell fraction were obtained after acquiring hundreds to thousands single-molecule events, consistent with previous studies. The minimal sample size were not predefined and the more molecule events the better the statistical significance. This is illustrated in Figure 4d. This plot shows that hundreds to thousands of single molecules are enough.
Data exclusions	No data exclusions
Replication	All experimental results were reproduced on multiple nanopore sensors. Overall, the work is comprised of >100,000 single-molecules events acquired on 15 nanopores.
Randomization	Randomization was not involved in this study. The workflow involved fabricating a nanopore, measuring a particular sample with the nanopore and analyzing the data.
Blinding	No blinding was used in thus study, since this is a proof-of-concept study to demonstrate the strategy for a digital scheme using nanopores and therefore did not require the stringency of blinding to validate the performance.

Reporting for specific materials, systems and methods

We require information from authors about some types of materials, experimental systems and methods used in many studies. Here, indicate whether each material, system or method listed is relevant to your study. If you are not sure if a list item applies to your research, read the appropriate section before selecting a response.

Materials & experimental systems

n/a	Involved in the study
[ ]	[x] Antibodies
[x]	[ ] Eukaryotic cell lines
[x]	[ ] Palaeontology and archaeology
[x]	[ ] Animals and other organisms
[x]	[ ] Human research participants
[x]	[ ] Clinical data
[x]	[ ] Dual use research of concern

Methods

n/a	Involved in the study
[x]	[ ] ChIP-seq
[x]	[ ] Flow cytometry
[x]	[ ] MRI-based neuroimaging

Antibodies

Antibodies used	Monoclonal anti human TSH beta subunit antibody (Maine Biotechnology Services, MAB130P), monoclonal anti human TSH beta subunit antibody (Fitzgerald Industries International, 10C-CR2151M4)
Validation	MAB130P: host mouse, isotype IgG kappa, clone ME130, affinity ~2.0 x 10^10 Litres/Mole<br>10C-CR2151M4: specificity human, host mouse, isotype IgG1, clone 157155, affinity ~2.0 x 10^10 Litres/Mole, TSH antibody was raised in mouse using human TSH beta subunit as the immunogen.
